# Modifying the Quality of Pig Carcasses, Meat, and Dry Fermented Sausage from Black Slavonian Pigs by Selecting the Final Body Weight and Nutrition

**DOI:** 10.3390/foods11091313

**Published:** 2022-04-30

**Authors:** Danijela Samac, Đuro Senčić, Zvonko Antunović, Josip Novoselec, Ivana Prakatur, Zvonimir Steiner, Željka Klir Šalavardić, Mario Ronta, Đurđica Kovačić

**Affiliations:** Faculty of Agrobiotechnical Sciences Osijek, Josip Juraj Strossmayer University of Osijek, V. Preloga 1, 31000 Osijek, Croatia; dsencic@fazos.hr (Đ.S.); zvonko.antunovic@fazos.hr (Z.A.); josip.novoselec@fazos.hr (J.N.); ivana.prakatur@fazos.hr (I.P.); zvonimir.steiner@fazos.hr (Z.S.); zeljka.klir@fazos.hr (Ž.K.Š.); mario.ronta@fazos.hr (M.R.); djkovacic@fazos.hr (Đ.K.)

**Keywords:** Black Slavonian pigs, feeding, final body weight, kulen, meat quality

## Abstract

A total of 96 Black Slavonian pigs were subjected to the research, in which they were split into 6 groups. Three groups (100, 120, and 130 kg) were fed a higher level (HL) of crude protein in fodder mixtures (CPFM), and three groups (100, 120, and 130 kg) were fed a lower level (LL) of CPFM. After the pigs were slaughtered, pig carcasses were dissected and the meat and halves quality indicators were determined. According to the influence of the final body weight (BW) and nutrition of pigs on the quality of their halves, meat, and dry fermented sausages (kulens), it was concluded that feeding an HL of CPFM increased the proportion of loin, belly rib part, and chin and increased the muscle tissue in the ham, loin, shoulder, neck, and belly rib parts. However, the chemical composition of the meat and the sensory properties of the kulen were not significantly affected by feeding the pigs an HL of CPFM. It was concluded that, by selecting the final BW and adjusting the feeding strategies for pigs, it is possible to modify the conformation and composition of pig carcasses and the quality of meat and kulens produced from the Black Slavonian pig, which is important because consumers prefer products with certain characteristics and of a standard quality and are ready to pay for them.

## 1. Introduction

The Black Slavonian pig, also known as “Fajferica”, is an autochthonous Croatian pig breed of the meaty and fatty type created at the end of the 19th century on the estate of Count Karl Pfeiffer called Orlovnjak, near Osijek. Count Pfeiffer mated Mangalica sows with Berkshire boars and then mated the offspring with Poland China boars [1]. From the end of the 19th century until the middle of the 20th century, the Black Slavonian pig was the most widespread pig breed in Croatia, but after the Second World War, because of modern, highly productive white-meat breeds from abroad, the breeding of this pig was neglected to the point where it almost became extinct [2]. However, due to good meat quality, resistance [3], longevity, and adaptability to extensive housing conditions [4,5], this breed was again recognized by Croatian producers, who realized that the Black Slavonian pig should be protected for biodiversity in a “bank of genes” that will permanently preserve genetic materials from rare and endangered breeds or individuals of the population [6]. This would encourage agricultural production, contributing to environmental protection, the preservation of biodiversity, and protection of rural areas [7]. Financial incentives provided by the government for the biological preservation of the breed cannot be used as a permanent solution. Therefore, this breed should be evaluated through adequate use for economic purposes.

To date, this breed has been grown extensively without systematic research on its production properties under the influence of various paragenetic factors, particularly nutrition. Taking similar European Mediterranean breeds (the Basque, the Gascon, and the Corsican pig in France; the Nero Siciliano and the Cinta Senese in Italy; and the Iberian pig in Spain) as a model, the Black Slavonian pig needs to be used for economic purposes by producing specific and protected products (fresh meat, dry fermented sausage, ham, greaves, etc.) with a trademark (brand) [8]. To preserve this breed, it is important to increase the production of traditional standardized meat products with a higher added value. Dry fermented sausage, referred to as kulen, is the product with the highest degree of valorization, price, and quality [9].

As a result of measures to halt the breed’s decline, research related to the Black Slavonian pig intensified at the beginning of the 21st century [4]. The effective population in 1996 was only 20 pigs, when the survival of the breed was endangered [10]. Croatia, therefore, signed the Biodiversity Treaty [11], and “A Survey of the State of Biological and Environmental Diversity of Croatia with Strategy and Protection Plan Action” was elaborated [12], as well as “A Program for Breeding up of the Black Slavonian Breed” [13]. Even today, the Black Slavonian pig is studied by few scientists from the region of its origin. Some research has been conducted on evaluating different housing and breeding systems [5,14], whereas several research groups have conducted various genetic studies [15,16,17,18] on breed determination and conservation of local breeds. Other studies have been related to the fattening of the Black Slavonian pig to obtain high-quality meat and meat products [19]. Mostly, the quality of pig halves, meat, kulen [20,21,22,23], ham [24], and sausages [25] has been studied.

The aim of this study is to show how selecting the final body weight (BW) of Black Slavonian pigs and feeding with different levels of crude protein in fodder mixtures (CPFM) influence the composition of pork halves (muscle, fat, and bone tissue), the conformation of pork halves (share of basic parts), and the composition of muscle tissue, particularly the content of intramuscular fat. Furthermore, the aim is to show that the modification of muscle tissue (meat) composition can improve the nutritional, physicochemical, and sensory properties of meat and kulen.

## 2. Materials and Methods

### 2.1. Animal Menagement

The study was conducted in compliance with the legal regulations set by the Animal Protection Act of Croatia (NN 133/06, NN 37/13, and NN 125/13) and the European Union Directive 2010/63/UE regarding animal protection, as approved by the Bioethics Committee for Research on Animals of the Faculty of Agrobiotechnical Sciences, Osijek (2158-94-02-22-01).

We used 96 Black Slavonian pigs, 32 of which were fattened up to approximately 100 kg final BW (groups A and B), 32 up to approximately 120 kg final BW (groups C and D), and 32 up to approximately 130 kg final BW (groups E and F). During the fattening, the pigs were fed fodder mixtures of grains (corn and barley) and a superconcentrate (Table 1). Groups A, C, and E were fed 14% CPFM during the first fattening period and 12% CPFM during the second fattening period, while groups B, D, and F were fed 12% CPFM during the first fattening period and 10% CPFM during the second fattening period. During the fattening, the pigs also consumed green alfalfa ad libitum. The sex ratio in each experimental group was equal (50:50%).

During the fattening, all pigs were kept in the same housing conditions, in a semi-outdoor system. After the fattening was completed, the pigs were transported to a slaughterhouse in a truck 24 h prior to being slaughtered. At the time of slaughter, the pigs were between 16 and 18 months old.

### 2.2. Carcass Measurements

After the pigs were slaughtered, pig carcasses were left to chill at +4 °C at a relative moisture of 90% for 24 h. After that, the right-half carcasses were dissected according to the modified method of [26].

The length of cold pig halves was measured by means of a measuring tape from *os pubis* to *atlas* and from *os pubis* to the 1st rib. Backfat thickness (S) was measured on the small of the back, at the point where *musculus gluteus medius pars piriformis* grows into bacon most intensively. The thickness of the longissimus lumborum in mm (M) was measured as the shortest connection between the front end of *musculus gluteus medius* and the upper edge of the vertebral canal. The surface area of the cross section (cm^2^) of *muscullus longissimus dorsi* (MLD) was measured between the 13th and 14th ribs, as per the method provided in [27], by means of a digital planimeter Haff Digiplan 305/306. The volume of the leg was measured at the widest part of the leg. A measuring tape was used to measure ham circumference and length.

### 2.3. Physico-Chemical Analysis of Meat

Meat quality indicators were investigated on a sample taken from the long back muscle (MLD), from between the 13th and 14th ribs. The pH_1_ value (45 min postmortem) was determined at the meat temperature of 35 °C, and the pH_2_ value (24 h postmortem) was measured after the meat was chilled at +4 °C. A contact pH meter (Mettler Toledo) was used for this purpose. The water-holding capacity (WHC) was determined by means of compression as per the method provided in [28], and consistency was presented as the surface area in cm^2^ of muscular tissue that was compressed on a filter paper in the process of measuring the WHC. Meat color was measured with a mobile device colorimeter Minolta Chromameter CR-410 (Minolta Camera Co., Ltd. Japan) Illuminant D65, Observer 2 degrees Closely matches CIE 1931Standard Observers: $\bar{x}$^2^λ, $\bar{y}$λ, $\overset{\cdot }{z}$λ, according to the standard CIE L* a* b* color system [29], according to the reference method in Honikel (1998), approved by the International Commission on Illumination CIE (1976). Meat color was measured on the long back muscle (MLD), taken from between the 13th and 14th ribs, cooled at +4 °C, after color stabilization (after 10 min), with 3 repetitions. The CPFM content in meat was determined according to the Kjeldahl method [30]. Intramuscular fat was determined according to the Soxhlet method [31]. The water content was defined as the loss sample of mass after the sample had been dried at 105 °C until constant mass was achieved. The ash content was determined by burning the organic matter at 550 °C until constant mass was achieved, and the ash content was shown as the percentage remains of the sample mass.

### 2.4. Kulen Menagement

After the carcass was dissected, meat from ham, loin, and shoulder, without fat and joint tissue, was separated for processing into kulens. The proportion (%) of the meat from ham and loin (80%) and shoulder (16.20%) was the same in all the tested groups. This “pure” muscular tissue was ground in a grinding machine (matrix diameter 6 mm and 8 mm), and the kulen mixture was prepared with 80% meat from ham and loin, 16.20% meat from shoulder, 2% salt, 1% sweet ground pepper, and 0.8% minced garlic. The mixture for each tested group of pigs was prepared separately and then stuffed into a pig cecum by means of a stuffing device. Thus prepared, the kulens were weighed and spread on drying racks in a drying room. For the first 15 days, the kulens were dried over smoke obtained by burning ash wood (*Fraxinus excelsior*). During these 15 days, fire was made 7 times. Kulens were then left to dry and mature for the next 9 months.

### 2.5. Kulen Analysis

After reaching maturity, the kulens were submitted for organoleptic evaluation to a taste panel consisting of 5 members with previous experience in assessment. The panelists have consented to participation in the study. Assessment was carried out at the Faculty of Agrobiotechnical Sciences Osijek. Each kulen sample was cut in half on a white glass plate with a knife. The assessor was provided one half to assess the appearance, the cross-section appearance, and the structure via visual inspection and touching. The assessor was provided the other half for tasting, for which each assessor was served a slice (around 0.5 cm thick) immediately after the kulen was cut. After every 6 evaluated kulens, the assessors took a break for an hour. During kulen assessment, the assessors were also served cheese, bread, apples, and water (at room temperature) to eliminate (neutralize) the traces of taste from the mouth between tasting individual samples. Kulen properties were scored as follows: appearance (1–5), structure (1–3), cross-section appearance (1–3), smell (1–5), taste (1–10), and general impression (1–5). A total of 8 kulens from each of the tested groups were assessed. The assessment of the sensory properties of the kulens was followed by an investigation of their physical and chemical properties.

The pH values of the kulen were measured with a contact pH meter (Mettler Toledo) applied to the center of the cross section. The kulen color was measured with a mobile device Minolta Chromameter CR-410 (Minolta Camera Co., Ltd. Japan) according to the standard CIE L* a* b* color system [29]. Water activity (a_w_) in the kulen was measured using a HygroLab 3 (Rotronic) by applying the Aw Quick model of operation on samples prepared by chopping and homogenizing 100 g of the central part of the kulen. The NaCl content in the kulen was determined by the titrimetric method [32], and the CPFM content of the kulen was obtained using the Kjeldahl method. Intramuscular fat was determined according to the Soxhlet method. The water content was defined as the loss of sample mass after the sample had been dried at 105 °C until constant mass was achieved. The ash content was determined by burning the organic matter at 550 °C until constant mass was achieved, and the ash content was shown as the percentage remains of the sample mass.

### 2.6. Statistical Analysis

Statistical data processing was performed by means of a Stat. Soft. Inc. (2012) computer software. Significance testing between and within groups was determined by an analysis of variance (ANOVA), and the calculated F value was compared with the theoretical F value. The significance of differences between mean values was determined using Fischer’s LSD test.

## 3. Results and Discussion

### 3.1. Indicators of the Quality of Pig Halves

The basic indicators of pig carcass quality are presented in Table 2. As the weight of the pig carcasses increased by weight groups, the length of the pig carcasses from os pubis to atlas also increased. The carcass length was similar to that of the Iberian pig [33] and the Majorcan Black pig [34]. For this property, a significant (*p* < 0.05) and a very significant difference (*p* < 0.01) were determined between the weight groups of pigs fed with a lower level (LL) of CPFM, whereas a very significant difference was found at a higher level (HL) of CPFM (*p* < 0.01) only between the 100 and 130 kg pig groups. Similar significant differences were determined also in terms of the carcass length from os pubis to the 1st rib.

As the pig carcass weight increased, so did the ham circumference. Therefore, very significant differences (*p* < 0.01) were determined among all weight groups of pigs fed with both lower and higher CPFM levels. The level of CPFM affected the ham volume only within the weight group of 100 kg pigs. Therefore, the hams of pigs fed with higher CPFM levels were very significantly (*p* < 0.01) higher in volume. The increase in the CPFM level in fodder mixtures affected the increase in ham length very significantly (*p* < 0.01) between the 120 and 130 kg pig groups, while in the 100 kg pig group, no significant difference (*p* > 0.005) was determined. The increase in the level of CPFM very significantly affected (*p* < 0.01) the increase in the ham index in the 120 and 130 kg pig groups. The increase in the BWs of pigs within groups fed with fodder mixtures with an LL of CP affected the increase in the ham index. An HL of CPFM affected the reduction in backfat thickness between the 120 and 130 kg pig groups. The increase in the BWs within pig groups fed with both LL and HL of CPFM led to a significant (*p* < 0.05) and a very significant (*p* < 0.01) increase in backfat thickness. Backfat thickness in the Black Slavonian pig is not excessive, unlike some other rural breeds. For example, in the research in [33], Iberian pigs of approximately 130 kg had a backfat thickness of 6.00 cm, whereas in the research in [35], even higher values were recorded for the Majorcan Black pig.

The level of CPFM did not significantly (*p* > 0.05) affect the cross-sectional area of MLD. However, this property was very significantly (*p* < 0.01) affected by the BWs of pigs, so that higher BW pigs had a very significantly (*p* < 0.01) smaller MLD area.

### 3.2. Conformation of Pig Carcasses

As can be seen from Table 3, no significant (*p* > 0.05) influence of the level of CPFM on the absolute proportion of hams in pig halves was determined, though a very significant (*p* < 0.01) or significant (*p* < 0.05) influence of the final BWs of pigs on the absolute proportion of hams was determined. In addition, [36] determined that the CPFM level in fodder mixtures had no effect on the absolute proportion of hams in pig halves. As the BWs increased within pig groups, the absolute proportion of hams increased. The level of CPFM did not affect the proportion of less valuable parts in the halves, but it affected very significantly (*p* < 0.01) the proportion of the loin, shoulder, neck, belly rib part, chin, and fat in some weight groups.

As the level of CPFM decreased, the loin proportion increased very significantly (*p* < 0.01) in all the weight groups, the shoulder proportion increased in the 120 kg pig group, and the neck and fat proportions increased in the 130 kg pig group. The proportion of belly rib part was significantly (*p* < 0.01) higher in all weight groups fed with fodder mixtures containing an HL of CP, which corresponds to previous studies by [36].

The relative proportions of basic parts in the halves are shown in Table 4. No significant (*p* > 0.05) impact of the level of CPFM or pig BW on the proportion of hams in the pig halves was found. In the research by [37], it was determined that pigs with lower final BWs produce carcasses with a significantly (*p* < 0.05) higher share of hams in halves.

With the increased level of CPFM, the proportion of the belly rib part increased very significantly (*p* < 0.01) in all the weight groups. The level of CPFM very significantly (*p* < 0.01) or significantly (*p* < 0.05) affected the proportion of other basic parts in pig halves in some weight groups. Thus, the neck and fat proportions were very significantly (*p* < 0.01) higher at an LL of CPFM in weight groups ranging from 100 to 130 kg and in the 130 kg pig group, respectively. As the BW increased, the relative proportion of loin also increased. Therefore, significant differences were determined between weight groups ranging from 100 to 130 kg at an HL of CPFM, between weight groups ranging from 100 to 130 kg at an LL of CPFM, as well as between weight groups ranging from 120 to 130 kg at an LL of CPFM. As the BW increased, the proportion of shoulder decreased significantly (*p* < 0.05) or very significantly (*p* < 0.01) at an HL, but not at an LL of CPFM. This was in accordance with the results obtained by [38], who investigated the effects of four dietary treatments characterized by ranging from 0 to 20% progressive reduction in the dietary CPFM and indispensable amino acid contents on the carcass quality and uniformity of pigs.

In the research on the influence of the final BW on the quality of pig carcasses of the Black Slavonian pigs, [19] observed that, as the final BW increased, the relative proportion of the shoulder in the carcasses decreased. The proportion of the belly rib part decreased significantly (*p* < 0.05) or very significantly (*p* < 0.01) with an increase in the pig BW within groups. Very significant differences (*p* < 0.01) were found between the 100 and 120 kg pig weight groups and between the 100 and 130 kg pig weight groups at an HL of CPFM, as well as between the 100 and 130 kg pig weight groups and between the 120 and 130 kg pig weight groups at an LL of CPFM. The chin proportion increased with increasing pig BW. Significant differences (*p* < 0.05) were determined between the 120 and 130 kg weight groups at an HL of CPFM and between the 100 and 120 kg pig weight groups, between the 100 and 130 kg pig weight groups, and between the 120 and 130 kg pig weight groups at an LL of CPFM. The fat proportion in the pig halves also increased with increasing pig BW, but very significant differences (*p* < 0.01) were found only between the 100 and 130 kg pig weight groups and between the 120 and 130 kg pig weight groups at an LL of CPFM. This was in accordance with the study in [37].

#### 3.2.1. The Tissue Proportion in Carcasses

As shown in Table 5, the increased level of CPFM affected the increase in the absolute and relative proportions of muscle tissue in halves in the same pig weight groups very significantly (*p* < 0.01), while no significant differences were found in terms of fat proportion (*p* > 0.05). In the study on the influence of CPFM levels on the composition of pig carcasses of Iberian pigs, [39] concluded that pigs fed a meal with an LL of CPFM significantly deposited fat in the carcass compared to pigs fed a meal with an HL of CP. Additionally, refs. [40,41] confirmed these conclusions with their research. Analogously, refs. [42,43] concluded that, when pigs are fed low-protein diets, fatter carcasses are produced compared with pigs fed with high-protein diets. However, they suggested the adoption of the net energy system and balanced amino acids as a means to still achieve acceptable performance, carcass characteristics, and meat quality.

As stated in the review paper by [44], heavier pigs are associated with a greater backfat thickness and a decreased percentage of fat-free or lean meat. This research also confirmed that an increase in the BWs of pigs had a very significant (*p* < 0.01) influence on the proportion of muscular and adipose tissue in pig carcasses among different weight groups. The absolute proportion of muscular tissue grew very significantly (*p* < 0.01) as the BW increased, whereas the relative proportion of muscular tissue decreased very significantly (*p* < 0.0.1) with the increase in BW within both levels of CPFM. Accordingly, the proportion of adipose tissue in carcasses grew very significantly (*p* < 0.01) as the BW increased within both levels of CPFM.

#### 3.2.2. Proportion of Muscular Tissue of the Basic Parts in the Weight of Pig Carcasses

As can be seen from Table 6, the increase in the level of CPFM had no significant (*p* > 0.05) influence on the increase in the muscular tissue proportion of ham in the 100 and 130 kg pig weight groups, whereas in the 120 kg pig weight group, a significantly (*p* < 0.05) higher proportion of the muscular tissue of ham was determined at an HL of CPFM.

As the BW of the pigs increased from 100 to 130 kg, a tendency was recorded toward a decrease in the proportion of the muscular tissue of a ham, but very significant differences (*p* < 0.01) were determined only between the 100 and 130 kg pig groups and between the 120 and 130 kg pig groups at an HL of CPFM. A significant difference (*p* < 0.05) was determined between the 100 and 130 kg pig groups at an LL of CPFM. A tendency toward an increase in the proportion of the muscular tissue of the loin was recorded with an increase in the CPFM content. However, a significant difference was determined only in the 130 kg pig weight group. An increase in the BW of pigs had no significant (*p* > 0.05) influence on the proportion of muscular tissue of the loin. Considering the proportion of shoulder and neck muscle tissue, no regular upward or downward trend was detected depending on the level of CPFM and the pig slaughter mass. A decrease in the level of CPFM led to a decrease in the proportion of muscular tissue in the belly rib part. Very significant (*p* < 0.01) differences in the part of this muscular tissue within the 120 and 130 kg pig weight groups were determined. An increase in the weight of the pig halves within the weight groups did not significantly (*p* > 0.05) affect the proportion of muscular tissue in the belly rib part at an HL of CPFM, but a very significant (*p* < 0.01) influence of the pig BW on this indicator at an LL of CPFM was determined.

#### 3.2.3. Proportion of Adipose Tissue of Basic Parts in the Weight of Pig Carcasses

As shown in Table 7, the proportion of CPFM did not significantly (*p* > 0.05) affect the proportion of adipose tissue of the ham in the weights of pig halves in the 100 and 120 kg pig weight groups, but a significant (*p* < 0.05) influence in the 130 kg pig weight group was determined.

An increase in the BW of pigs by weight groups led to the tendency for an increase in the proportion of adipose tissue of ham, but very significant differences (*p* < 0.01) were detected only between the 100 and 130 kg pig groups and between the 120 and 130 kg pig groups at an HL of CPFM, and significant differences (*p* < 0.05) were detected between the 100 and 120 kg weight groups at an LL of CPFM.

The proportion of adipose tissue of the loin was higher at an LL of CPFM in all pig weight groups, but very significant differences (*p* < 0.01) were detected only within the 100 and 130 kg weight groups. The increase in the BWs of pigs led to the tendency of increase in the proportion of adipose tissue of the loin in all weight groups at both LL and HL of CPFM. Very significant differences (*p* < 0.01) were determined between the 100 and 120 kg weight groups and between the 100 and 130 kg weight groups at an HL of CPFM and also between the 120 and 130 kg weight groups at an LL of CPFM. The proportion of adipose tissue of the neck was higher at an LL of CPFM in all pig weight groups, but a significant difference (*p* < 0.05) was determined within the 100 kg weight group.

As the pig carcass weight increased, the relative proportion of adipose tissue of the neck in all pig weight groups at both LL and HL of CPFM also increased, but a very significant difference (*p* < 0.01) was determined only between the 100 and 120 kg weight groups and a significant difference (*p* < 0.05) was determined between the 100 and 130 kg weight groups at an HL of CPFM. The proportion of adipose tissue of the belly rib part was higher at an HL of CPFM in all the pig weight groups, but very significant differences (*p* < 0.01) were detected only in the 100 kg weight group. With the increase in pig BW, the relative proportion of adipose tissue of the belly rib part decreased at an HL of CPFM, while at an LL of CPFM, it increased. Very significant differences (*p* < 0.01) were detected between the 100 and 120 kg pig weight groups at an HL of CPFM, between the 100 and 130 kg pig weight groups at an HL of CPFM, and between the 100 and 130 kg weight groups at an LL of CPFM. The proportion of adipose tissue of the chin was higher in the 100 kg weight group at an HL of CPFM. The proportion of adipose tissue of fat was higher at an LL of CPFM in the 130 kg weight group, whereas in all the other weight groups, no significant differences (*p* > 0.05) were detected in terms of the CPFM level. Moreover, no influence of pig BW on the relative proportion of adipose tissue of fat was determined.

#### 3.2.4. Proportion of Bone Tissue of the Basic Parts in the Weight of Pig Carcasses

The proportion of bone tissue of the ham in the weight of pig carcasses did not significantly (*p* > 0.05) differ between pig groups, as well as in terms of the level of CPFM and pig BW (Table 8).

### 3.3. Pork Quality Indicators

#### 3.3.1. Physico-Chemical Properties of Meat

The physical properties of the meat are shown in Table 9. The pH value of the meat is an important characteristic of meat quality, as the transformation of muscles into meat changes its pH value. A neutral pH value shifts toward an acidic pH value. According to the classification of [45,46], the preferred values for pH_1_ are above 6.0 (a value between 5.8 and 6.0 is suspicious), while for pH_2_, values below 5.7 indicate PSE meat (i.e., pale, soft, and exudative meat) and values above 6.0 indicate DFD meat (i.e., dark, firm, and dry meat) [47].

The data obtained for the pH_1_ and pH_2_ values, WHC, and meat consistency point to meat of normal quality, and no significant differences (*p* > 0.05) were determined among the groups of pigs when considering their final BWs and the level of CPFM. According to [48], pigs fed with an LL of CPFM have meat with a lower pH_1_ value and WHC, while [41] reported the limited influence of the level of CPFM on the meat’s pH value. In [49], a decrease in WHC was observed when pigs were fed reduced CPFM levels. As per [50], as the BWs of pigs increased from 100 to 160 kg, the pH values of the meat decreased. This correlates with the results obtained by [51], who determined higher pH values of meat in pigs of lower weight. For white modern breeds, [3] determined that an increase in the BWs of pigs was also followed by an increase in the pH values, WHC, and marbling.

Surface color is an important visual quality indicator of meat, and meat color measurements are usually expressed on the L* (lightness), a* (redness), and b* (yellowness) scale [52]. An increase in the level of CPFM did not significantly (*p* > 0.05) influence differences in terms of the L* value for meat color in the 100 and 120 kg weight groups, but a significant difference (*p* < 0.05) in the 130 kg weight group was detected. In this research, in most of the investigated groups of pigs, the L* values for meat somewhat exceeded the desired values (43–50) reported by [53]. Higher L* values (HL of lightness) are a consequence of a higher proportion of fat in the meat, which ranged from 6.97% in group 1 to as much as 16.98% in group 6. According to [48], pigs fed with lower-CPFM diets have a lighter (L*) meat color. In terms of the level of CPFM, [41] determined a limited effect of diet on meat color. As the BWs increased within weight groups at an HL of CPFM, no significant differences (*p* < 0.05) were determined for this meat quality between the 120 and 130 kg pig groups and a very significant difference (*p* < 0.01) was determined between the 100 and 130 kg pig groups at an LL of CPFM. In [54], it was indicated that the higher final BW of pigs very significantly (*p* < 0.01) influenced the L* value for meat color, whereas [51] indicated that an increase in the L* value for meat color corresponds to a final BW increase. Many authors [55,56,57] have not recorded any statistically significant differences (*p* > 0.05) in the L* values for meat color in relation to pig BW.

Very significant differences (*p* < 0.01) were determined between pig groups with regard to the level of CPFM for a* values of meat color. With an increase in pig BW by weight groups at an HL of CP, no significant differences (*p* > 0.05) for a* values were detected, while very significant differences (*p* < 0.01) were determined for this parameter between the 100 and 130 kg pig weight groups and between the 120 and 130 kg pig weight groups at an LL of CPFM. In [54], it was indicated that higher final BWs of pigs very significantly (*p* < 0.01) influenced the a* value for meat color. As per [58] also, there were significant differences (*p* < 0.05) in the level of meat redness between the 100 and 130 kg pigs. In the research carried out by [59], higher a* values for meat color were determined in heavier pigs, whereas [56,57] did not determine statistically significant differences in terms of the a* value for meat color. Increased a* values for meat color, which were recorded within pigs with higher final BWs, are in correlation with a higher content of muscular pigment in older pigs.

In terms of the b* values of meat color, no significant differences (*p* > 0.05) were detected between the 100 and 120 kg pig groups and with an increased level of CPFM. As the BWs increased by weight groups at an HL of CPFM, no significant differences (*p* > 0.05) between the 100 and 130 kg pig weight groups and between the 120 and 130 kg pig weight groups were detected, whereas significant differences (*p* < 0.05) were detected between the 100 and 130 kg pig weight groups and between the 120 and 130 kg pig weight groups at an LL of CPFM by weight groups. In our previous research [37], higher b* values were determined for the meat color of heavier animals. While [54] determined that higher final BW of pigs very significantly (*p* < 0.01) influenced the b* value for color, [55,56,57] did not find any statistically significant differences in terms of the b* value for meat color.

#### 3.3.2. Basic Chemical Properties of Meat

As can be seen from Table 10, with an increased level of CPFM, the water and protein content in meat also increased within the weight groups of pigs, but not statistically significantly (*p* > 0.05). As expected, the water and protein content in the meat decreased very significantly (*p* < 0.01) as the weight of the carcasses increased by weight groups between the 100 and 120 kg weight groups and between the 100 and 130 kg weight groups at both LL and HL of CP. This is in agreement with our previous studies [19,37], in which we determined that pigs with lower BWs produce meat with a higher water and protein content. However, as the level of CPFM increased, the fat content in the meat decreased within all pig weight groups, but not in a statistically significant way (*p* > 0.05). With the increase in the weights of pig halves within weight groups, the fat content also increased very significantly (*p* < 0.01) between the 100 and 120 kg pig weight groups and between the 100 and 130 kg pig weight groups at both LL and HL of CPFM, whereas between the 120 and 130 kg weight groups, no significant differences (*p* > 0.05) were detected.

With the increase in the level of CPFM, no statistically significant differences (*p* > 0.05) in the ash content were detected within weight groups. As the weight of carcasses increased by weight groups, very significant differences (*p* < 0.01) were detected in the ash content of meat between the 100 and 120 kg pig weight groups and between the 100 and 130 kg pig weight groups at both LL and HL of CP, whereas between the 120 and 130 kg weight groups, no significant differences (*p* > 0.05) were detected. As assumed, an increase in the final BWs of pigs caused an increase in the content of intramuscular fat and a decrease in the content of CPFM and water in meat.

### 3.4. Indicators of Kulen Quality

#### 3.4.1. Physical and Chemical Properties of Kulen

In terms of the pH value of kulens, kulen color (L*, a*, and b* values), and the content of NaCl and water in kulens, no significant differences (*p* > 0.05) were detected among groups when taking into consideration the level of CPFM and the BWs of pigs. Kulens with slightly lower pH values were found in the research of [60] (5.35) and [61] (5.07–5.75) if compared to the pH values of kulens obtained in this research (5.86–5.97), which can be attributed to the influence of different pig genotypes, different production technologies, and different phases of kulen ripening. These studies were carried out on Slavonian kulens produced by various producers and originating from different areas of Slavonia. As expected, with an increase in the level of CPFM within weight groups, the protein content in kulens also increased, but not in a statistically significant way (*p* > 0.05). As the weights of pig halves by weight groups increased, the protein content in kulens decreasing very significantly (*p* < 0.01) between the 100 and 120 kg pig groups and between the 100 and 130 kg pig groups at both HL and LL of CP. No significant differences were detected (*p* > 0.05) between the 120 and 130 kg pig weight groups in terms of the protein content in kulens. The research results for the protein content in kulens obtained by [60] (22.92%), [61] (30.3–39.6), and [20] (40.99%) as well as the results of this research (43.59–45.94%) indicate that kulens, compared to some other traditional sausages researched by other authors [62,63], have a higher average protein content. An increased level of CPFM did not significantly (*p* > 0.05) affect the fat content in kulens within the 120 and 130 kg pig weight groups, whereas in the 100 kg weight group, a very significant difference (*p* < 0.01) was determined, because pigs fed with an HL of CPFM gave kulens with less fat. The final BWs of pigs had a very significant (*p* < 0.01) influence on the fat content in kulens between the 100 and 120 kg pig groups and between the 100 and 130 kg pig groups with an HL of CP and between the 100 and 120 kg pig groups with an LL of CP. Increased BWs of pigs resulted in increased crude fat in kulens. Compared to the results obtained by [60] (24.23–60.34%), [61] (16.40–31.00%), and [20] (23.03%), in this research, a lower fat content in kulens was determined (16.24–18.51%). This can be explained by differences in the production of kulens in their research (fatter meat, backfat added, etc.). No statistically significant differences (*p* > 0.05) were detected in terms of ash, water, and NaCl content, as well as L*, a*, b*, and a_w_ values in kulens among the groups of pigs of different weights. The results of the research on the physico-chemical properties of kulens are in line with expectations and the results of the chemical analysis of meat from Black Slavonian pig (Table 11), which indicate that, with increasing levels of CPFM, the crude fat content decreases, while the CPFM content in meat increases among pig weight groups, but not in a statistically significant way (*p* > 0.05).

#### 3.4.2. Sensory Properties of Kulens

The sensory properties of kulens are provided in Table 12. No significant differences (*p* > 0.05) were recorded in terms of appearance (it was even), structure (firm, but not too hard), cross-section appearance (it was uniform in all groups), and smell (it was nice) between the groups when considering the level of CPFM and the final BWs of pigs. An increase in the level of CPFM did not significantly (*p* > 0.05) affect the taste of the kulens. However, the BW before slaughter significantly influenced the taste of the kulens. Pigs with higher BWs provided better-tasting kulens. Very significant differences (*p* < 0.01) were detected in terms of the taste between kulens produced from pigs with BWs of 100 and 120 kg and from pigs with BWs of 100 and 130 kg at an HL of CPFM and between the 100 and 120 kg pig groups and between the 100 and 130 kg pig groups at an LL of CPFM.

It should be emphasized that the Black Slavonian pigs from all the investigated weight groups (100, 120, and 130 kg) resulted in the production of good-quality kulens. The reason for this is the already “mature” meat in pigs with BWs of 100 kg, as they have a high fat content, i.e., a higher dry matter content. Another reason is that, compared to modern high-meat genotype pigs, the Black Slavonian pigs reach that BW when they are older and they also have an adequate enzyme composition of meat.

## 4. Conclusions

An HL of CPFM significantly increased the absolute and relative proportions of the loin, the chin, and the belly rib part and decreased the less valuable part proportion in all weight groups. If compared to pigs fed with an LL of CPFM, a significantly higher proportion of muscular tissue was determined in the hams, loins, shoulders, necks, and belly rib parts of pigs fed a meal with an HL of CPFM. An increase in the final BWs led to an increase in the absolute proportions of the ham, loin, chin, adipose tissue, and less valuable parts as well as to an increase in the relative proportions of the loin and adipose tissue within all weight groups. No significant differences were detected in terms of pH value, kulen color values (L*, a*, and b*), and the content of NaCl, water, and ash in kulens, considering the final BWs of pigs, but it was determined that, as the final BWs of pigs increased, the CPFM content in kulens decreased very significantly. The level of CPFM did not significantly affect the sensory properties of kulens, but the increase in pig BW very significantly improved kulen taste.

In general, by selecting the final BWs of pigs and specific feeding strategies for pigs, it is possible to modify the conformation and composition of pig carcasses and the quality of meat and kulens produced from the Black Slavonian pig. The obtained results are important because consumers prefer meat and meat products of specific characteristics and a standard quality and are ready to pay for them and, by selecting preferred meat characteristics, producers can ensure this.

## Figures and Tables

**Table 1:** The mixing ratio of grains and superconcentrate “35” in the experimental fattening.

Fattening Period	Fodder Mixture	Proportion (%)	CPFM Proportion (%)	Metabolic Energy (MJ/kg)
HL of CP				
1st (25−60 kg)	Corn	78.00	6.31	
	Super “35”	22.00	7.70	
		100.00	14.01	13.37
2nd (60−100, 120, or 130 kg)	Corn	68.00	5.54	
	Barley	20.00	2.14	
	Super “35”	12.00	4.20	
		100.00	11.88	13.34
LL of CP				
1st (25−60 kg)	Corn	85.00	6.88	
	Super “35”	15.00	5.25	
		100.00	12.13	13.30
2nd (60−100, 120, or 130 kg)	Corn	70.00	5.67	
	Barley	25.00	2.67	
	Super “35”	5.00	1.75	
		100.00	10.09	13.25

HL of CP—high level of crude protein; LL of CP—lower level of crude protein.

**Table 2:** Basic indicators of the quality of pig carcasses.

Indicators	A	B	C	D	E	F
	$\bar{x}$ ± s	$\bar{x}$ ± s	$\bar{x}$ ± s	$\bar{x}$ ± s	$\bar{x}$ ± s	$\bar{x}$ ± s
Pig BW (kg)	100.62 ^A^ ± 1.36	100.75 ^a^ ± 1.24	120.37 ^B^ ± 1.36	121.12 ^b^ ± 2.02	131.00 ^C^ ± 1.63	131.37 ^c^ ± 0.88
Cold carcass mass (kg)	38.30 ^A^ ± 1.12	37.07 ^a^ ± 0.56	48.06 ^B^ ± 0.95	47.85 ^b^ ± 0.58	53.43 ^C^ ± 1.11	52.93 ^c^ ± 0.36
Half length (cm) Os pubis−atlas	97.19 ^A^ ± 3.45	95.25 ^a^ ± 1.73	101.12 ^AB^ ± 1.20	100.25 ^b^ ± 1.87	104.37 ^B^ ± 1.15	106.62 ^c^ ± 9.32
Half length (cm), Os pubis−1st rib	78.25 ^A^ ± 2.74	78.19 ^a^ ± 2.71	85.31 ^B^ ± 1.40	95.65 ^b^ ± 13.45	84.31 ^xAB^ ± 1.96	93.69 ^yb^ ± 4.44
Volume of leg (cm)	64.87 ^xA^ ± 1.09	62.56 ^ya^ ± 2.34	67.12 ^B^ ± 1.20	68.25 ^b^ ± 2.32	70.94 ^C^ ± 1.06	72.12 ^c^ ± 1.84
Leg length (cm)	27.19 ^A^ ± 2.34	26.50 ^a^ ± 1.86	28.50 ^xAB^ ± 0.89	34.94 ^yb^ ± 5.30	30.19 ^xB^ ± 1.90	40.69 ^yc^ ± 0.48
Fat thickness (cm)	3.30 ^A^ ± 0.37	3.00 ^a^ ± 0.53	3.66 ^xA^ ± 0.50	4.26 ^yb^ ± 0.37	4.85 ^xB^ ± 0.21	5.53 ^yc^ ± 0.43
Sectional area MLD-a (cm^2^)	34.62 ^A^ ± 0.72	34.50 ^a^ ± 1.03	33.12 ^B^ ± 0.96	32.87 ^b^ ± 0.96	32.62 ^B^ ± 1.86	32.25 ^b^ ± 1.24

^x,y^—different letters differ significantly (*p* < 0.05) within the final body weight level group (100 kg, 120 kg, and 130 kg). ^A,B,C^—different letters differ significantly (*p* < 0.05) between 1, 3, and 5 groups (fed with 14% CPFM during the first fattening period and with 12% CPFM during the second fattening period). ^a,b,c^—different letters differ significantly (*p* < 0.05) between 2, 4, and 6 groups (fed with 12% CPFM during the first fattening period and with 10% CPFM during the second fattening period).

**Table 3:** Shares of the basic parts of the pig carcasses (kg).

Part of Half	Groups of Pigs					
	A	B	C	D	E	F
	$\bar{x}$ ± s	$\bar{x}$ ± s	$\bar{x}$ ± s	$\bar{x}$ ± s	$\bar{x}$ ± s	$\bar{x}$ ± s
Ham (kg)	9.75 ^A^ ± 0.35	9.69 ^a^ ± 1.23	12.82 ^B^ ± 0.93	12.52 ^b^ ± 0.61	13.64 ^C^ ± 0.44	13.39 ^c^ ± 0.47
Loin (kg)	5.16 ^xA^ ± 0.30	6.27 ^ya^ ± 0.76	7.60 ^xB^ ± 0.78	8.58 ^yb^ ± 1.11	8.90 ^xC^ ± 1.37	11.23 ^yc^ ± 0.39
Shoulder (kg)	5.87 ^xA^ ± 0.27	4.82 ^ya^ ± 0.35	5.53 ^xA^ ± 0.65	6.45 ^yb^ ± 0.66	6.78 ^B^ ± 0.57	6.78 ^b^ ± 0.23
Neck (kg)	2.78 ^A^ ± 0.25	4.27 ^a^ ± 1.48	5.52 ^B^ ± 1.85	5.44 ^a^ ± 1.35	4.78 ^xC^ ± 1.27	6.79 ^yb^ ± 0.32
Belly rib part (kg)	9.36 ^xA^ ± 0.41	6.72 ^yab^ ± 1.37	9.96 ^xA^ ± 0.47	7.57 ^ya^ ± 2.68	11.32 ^xB^ ± 0.44	6.11 ^yb^ ± 0.15
Chin (kg)	1.12 ^xA^ ± 0.18	0.79 ^ya^ ± 0.24	1.13 ^A^ ± 0.31	1.36 ^b^ ± 0.34	1.57 ^B^ ± 0.32	1.48 ^b^ ± 0.16
Fat (kg)	1.28 ^A^ ± 0.16	1.08 ^a^ ± 0.23	1.63 ^B^ ± 0.45	1.51 ^b^ ± 0.38	2.11 ^xC^ ± 0.45	2.59 ^yc^ ± 0.17
Less valuable parts (kg)	2.96 ^A^ ± 0.11	3.43 ^a^ ± 0.69	3.87 ^B^ ± 0.40	4.42 ^b^ ± 0.96	4.33 ^B^ ± 0.59	4.56 ^b^ ± 0.36

^x,y^—different letters differ significantly (*p* < 0.05) within the final body weight level group (100 kg, 120 kg, and 130 kg). ^A,B,C^—different letters differ significantly (*p* < 0.05) between 1, 3, and 5 groups (fed with 14% CPFM during the first fattening period and with 12% CPFM during the second fattening period). ^a,b,c^—different letters differ significantly (*p* < 0.05) between 2, 4, and 6 groups (fed with 12% CPFM during the first fattening period and with 10% CPFM during the second fattening period).

**Table 4:** Shares of the basic parts of the pig carcasses (%).

Part of Half	Groups of Pigs					
	A	B	C	D	E	F
	$\bar{x}$ ± s	$\bar{x}$ ± s	$\bar{x}$ ± s	$\bar{x}$ ± s	$\bar{x}$ ± s	$\bar{x}$ ± s
Ham (%)	25.45 ± 0.60	26.11 ± 1.41	26.66 ± 1.70	26.17 ± 1.09	25.53 ± 0.84	25.30 ± 0.95
Loin (%)	13.46 ^A^ ± 0.69	15.50 ^a^ ± 6.09	15.82 ^AB^ ± 1.69	17.92 ^a^ ± 2.18	16.66 ^B^ ± 2.58	21.22 ^b^ ± 0.73
Shoulder (%)	15.33 ^xA^ ± 0.62	13.10 ^y^ ± 1.02	11.53 ^xB^ ± 1.49	13.48 ^y^ ± 1.35	12.67 ^C^ ± 0.84	12.80 ± 0.35
Neck (%)	7.26 ^xA^ ± 0.65	12.29 ^y^ ± 2.72	11.45 ^B^ ± 3.75	11.38 ± 2.86	8.97 ^xAB^ ± 2.47	12.83 ^y^ ± 0.52
Belly rib part (%)	24.45 ^xA^ ± 0.99	18.09 ^ya^ ± 2.76	20.74 ^xB^ ± 1.09	15.83 ^ya^ ± 5.63	21.19 ^xB^ ± 0.81	11.53 ^yb^ ± 0.22
Chin (%)	2.95 ^xAB^ ± 0.38	2.17 ^ya^ ± 0.70	2.34 ^A^ ± 0.65	2.84 ^b^ ± 0.71	2.95 ^B^ ± 0.55	2.81 ^b^ ± 0.32
Fat (%)	3.36 ± 0.35	2.90 ^a^ ± 0.47	3.39 ± 0.96	3.14 ^a^ ± 0.80	3.94 ^x^ ± 0.79	4.90 ^yb^ ± 0.33
Less valuable parts (%)	7.74 ^x^ ± 0.26	9.84 ^y^ ± 1.03	8.07 ^x^ ± 0.78	9.24 ^y^ ± 0.07	8.09 ± 1.20	8.61 ± 0.66

^x,y^—different letters differ significantly (*p* < 0.05) within the final body weight level group (100 kg, 120 kg, and 130 kg). ^A,B,C^—different letters differ significantly (*p* < 0.05) between 1, 3, and 5 groups (fed with 14% CPFM during the first fattening period and with 12% CPFM during the second fattening period). ^a,b,c^—different letters differ significantly (*p* < 0.05) between 2, 4, and 6 groups (fed with 12% CPFM during the first fattening period and with 10% CPFM during the second fattening period).

**Table 5:** Shares of tissue in the carcass.

Type of Tissue	Groups of Pigs					
	A	B	C	D	E	F
	$\bar{x}$ ± s	$\bar{x}$ ± s	$\bar{x}$ ± s	$\bar{x}$ ± s	$\bar{x}$ ± s	$\bar{x}$ ± s
Muscle tissue kg	18.55 ^xA^ ± 0.90	17.14 ^ya^ ± 1.77	22.78 ^xB^ ± 1.29	20.71 ^yb^ ± 1.36	22.65 ^xB^ ± 0.64	20.53 ^yb^ ± 1.30
%	48.47 ^A^ ± 2.57	46.29 ^a^ ± 1.88	47.37 ^xA^ ± 2.52	43.29 ^yb^ ± 2.93	42.39 ^xB^ ± 1.76	38.76 ^yc^ ± 2.21
Adipose tissue kg	13.68 ^A^ ± 1.23	12.87 ^a^ ± 1.76	17.08 ^B^ ± 1.85	18.29 ^b^ ± 1.80	22.19 ^C^ ± 2.05	23.08 ^c^ ± 1.52
%	35.69 ^A^ ± 2.62	34.69 ^a^ ± 2.09	35.55 ^A^ ± 4.00	38.21 ^b^ ± 3.55	41.49 ^B^ ± 3.27	43.64 ^c^ ± 3.16
Bone tissue kg	3.09 ^A^ ± 0.12	3.63 ± 0.60	4.34 ^B^ ± 0.53	4.43 ± 0.67	4.26 ± 0.47	4.76 ^B^ ± 0.66
%	8.10 ^x^ ± 0.26	9.82 ^y^ ± 0.96	9.01 ± 0.99	9.26 ± 1.40	8.03 ± 0.97	8.99 ± 1.21

^x,y^—different letters differ significantly (*p* < 0.05) within the final body weight level group (100 kg, 120 kg, and 130 kg). ^A,B,C^—different letters differ significantly (*p* < 0.05) between 1, 3, and 5 groups (fed with 14% CPFM during the first fattening period and with 12% CPFM during the second fattening period). ^a,b,c^—different letters differ significantly (*p* < 0.05) between 2, 4, and 6 groups (fed with 12% CPFM during the first fattening period and with 10% CPFM during the second fattening period).

**Table 6:** Shares of muscle tissue of the basic parts in the weight of the pig carcasses.

Muscle Tissue of the Basic Parts of Halves	Groups of Pigs					
	A	B	C	D	E	F
	$\bar{x}$ ± s	$\bar{x}$ ± s	$\bar{x}$ ± s	$\bar{x}$ ± s	$\bar{x}$ ± s	$\bar{x}$ ± s
Ham (%)	15.57 ^A^ ± 0.97	15.19 a ± 1.31	15.58 ^xA^ ± 1.95	14.11 ^yab^ ± 1.40	12.85 ^xB^ ± 1.05	13.62 ^yb^ ± 0.72
Loin (%)	6.93 ± 0.87	7.15 ± 1.61	6.70 ± 0.62	7.36 ± 1.35	6.38 ^x^ ± 0.86	7.94 ^y^ ± 0.37
Shoulder (%)	9.58 ^xA^ ± 0.38	7.68 ^ya^ ± 0.85	6.66 ^xB^ ± 1.07	7.93 ^ya^ ± 0.62	7.08 ^B^ ± 0.38	6.87 ^b^ ± 0.63
Neck (%)	4.92 ^A^ ± 0.36	6.31 ^a^ ± 1.42	7.15 ^B^ ± 2.10	6.87 ^ab^ ± 1.65	5.28 ^xA^ ± 1.23	7.79 ^yb^ ± 0.74
Belly rib part (%)	11.47 ± 0.93	9.94 ^a^ ± 1.33	11.28 ^x^ ± 1.02	7.00 ^yb^ ± 4.29	10.79 ^x^ ± 0.20	2.55 ^yc^ ± 0.08

^x,y^—different letters differ significantly (*p* < 0.05) within the final body weight level group (100 kg, 120 kg, and 130 kg). ^A,B,C^—different letters differ significantly (*p* < 0.05) between 1, 3, and 5 groups (fed with 14% CPFM during the first fattening period and with 12% CPFM during the second fattening period). ^a,b,c^—different letters differ significantly (*p* < 0.05) between 2, 4, and 6 groups (fed with 12% CPFM during the first fattening period and with 10% CPFM during the second fattening period).

**Table 7:** Shares of adipose tissue of the basic parts in the weight of the pig carcasses.

Adipose Tissue of the Basic Parts of Halves	Groups of Pigs					
	A	B	C	D	E	F
	$\bar{x}$ ± s	$\bar{x}$ ± s	$\bar{x}$ ± s	$\bar{x}$ ± s	$\bar{x}$ ± s	$\bar{x}$ ± s
Ham (%)	7.47 ^A^ ± 0.96	8.12 ^a^ ± 0.54	8.40 ^A^ ± 0.95	9.44 ^b^ ± 1.19	10.33 ^xB^ ± 1.27	9.03 yb ± 1.40
Loin (%)	4.93 ^xA^ ± 0.33	7.75 ^ya^ ± 1.40	7.29 ^B^ ± 1.58	8.32 ^a^ ± 1.19	8.51 ^xB^ ± 2.06	10.98 ^yb^ ± 0.58
Shoulder (%)	4.17 ^A^ ± 0.52	3.68 ± 0.51	3.24 ^B^ ± 0.86	3.78 ± 0.94	4.11 ^A^ ± 0.54	4.25 ± 0.40
Neck (%)	1.30 ^xA^ ± 0.27	3.20 ^y^ ± 1.23	2.72 ^B^ ± 1.34	2.86 ± 0.78	2.53 ± 0.98	3.42 ^B^ ± 0.63
Belly rib part (%)	11.51 ^xA^ ± 1.04	6.85 ^ya^ ± 1.44	8.16 ^B^ ± 0.68	7.81 ^ab^ ± 1.35	9.14 ^B^ ± 0.70	8.26 ^b^ ± 0.15
Chin (%)	2.94 ^xAB^ ± 0.38	2.17 ^ya^ ± 0.70	2.34 ^A^ ± 0.65	2.85 ^b^ ± 0.71	2.95 ^B^ ± 0.55	2.81 ^b^ ± 0.32
Fat (%)	3.36 ± 0.35	2.90 ^a^ ± 0.47	3.39 ± 0.96	3.14 ^a^ ± 0.80	3.94 ^x^ ± 0.79	4.89 ^yb^ ± 0.33

^x,y^—different letters differ significantly (*p* < 0.05) within the final body weight level group (100 kg, 120 kg, and 130 kg). ^A,B^—different letters differ significantly (*p* < 0.05) between 1, 3, and 5 groups (fed with 14% CPFM during the first fattening period and with 12% CPFM during the second fattening period). ^a,b^—different letters differ significantly (*p* < 0.05) between 2, 4, and 6 groups (fed with 12% CPFM during the first fattening period and with 10% CPFM during the second fattening period).

**Table 8:** Shares of bone tissue of the basic parts in the weight of pig carcasses.

Bone Tissue of the Basic Parts of Halves	Groups of Pigs					
	A	B	C	D	E	F
	$\bar{x}$ ± s	$\bar{x}$ ± s	$\bar{x}$ ± s	$\bar{x}$ ± s	$\bar{x}$ ± s	$\bar{x}$ ± s
Ham (%)	2.41 ± 0.08	2.80 ± 0.40	2.68 ± 0.36	2.61 ± 0.45	2.34 ± 0.41	2.65 ± 0.51
Loin (%)	1.60 ^x^ ± 0.22	2.22 ^y^ ± 0.29	1.82 ^x^ ± 0.36	2.24 ^y^ ± 0.46	1.77 ^x^ ± 0.08	2.30 ^y^ ± 0.06
Shoulder (%)	1.57 ± 0.07	1.73 ± 0.13	1.63 ± 0.18	1.75 ± 0.27	1.49 ± 0.10	1.69 ^y^ ± 0.27
Neck (%)	1.04 ^x^ ± 0.10	1.78 ^y^ ± 0.52	1.58 ± 0.46	1.65 ± 0.52	1.16 ± 0.35	1.62 ± 0.54
Belly rib part (%)	1.47 ^A^ ± 0.23	1.29 ^a^ ± 0.23	1.30 ^AB^ ± 0.18	1.00 ^b^ ± 0.24	1.24 ^B^ ± 0.31	0.72 ^c^ ± 0.04

^x,y^—different letters differ significantly (*p* < 0.05) within the final body weight level group (100 kg, 120 kg, and 130 kg). ^A,B,C^—different letters differ significantly (*p* < 0.05) between 1, 3, and 5 groups (fed with 14% CPFM during the first fattening period and with 12% CPFM during the second fattening period). ^a,b,c^—different letters differ significantly (*p* < 0.05) between 2, 4, and 6 groups (fed with 12% CPFM during the first fattening period and with 10% CPFM during the second fattening period).

**Table 9:** Physical properties of meat.

Indicators	Groups of Pigs					
	A	B	C	D	E	F
	$\bar{x}$ ± s	$\bar{x}$ ± s	$\bar{x}$ ± s	$\bar{x}$ ± s	$\bar{x}$ ± s	$\bar{x}$ ± s
pH_1_	6.72 ± 0.03	6.74 ± 0.03	6.75 ± 0.03	6.72 ± 0.03	6.71 ± 0.04	6.71 ± 0.04
pH_2_	5.81 ± 0.05	5.79 ± 0.05	5.80 ± 0.04	5.79 ± 0.06	5.79 ± 0.04	5.78 ± 0.03
Water-holding capacity (cm^2^)	4.34 ± 0.04	4.34 ± 0.02	4.35 ± 0.03	4.33 ± 0.04	4.34 ± 0.02	4.33 ± 0.03
Consistency (cm^2^)	2.47 ± 0.12	2.51 ± 0.14	2.49 ± 0.14	2.46 ± 0.10	2.50 ± 0.10	2.45 ± 0.11
Color (CIE L*)	51.74 ± 1.96	55.10 ^a^ ± 3.74	53.75 ± 3.25	52.75 ^a^ ± 5.08	52.80 ^x^ ± 1.74	48.99 ^yb^ ± 0.76
Color (CIE a*)	17.30 ^x^ ± 0.70	18.90 ^ya^ ± 0.77	18.43 ^x^ ± 1.36	19.91 ^ya^ ± 1.68	18.28 ^x^ ± 0.80	23.02 ^yb^ ± 1.22
Color (CIE b*)	4.58 ^A^ ± 0.80	5.74 ^a^ ± 0.91	5.80 ^B^ ± 1.06	5.67 ^a^ ± 1.08	5.08 ^xAB^ ± 0.87	6.86 ^yb^ ± 1.04

^x,y^—different letters differ significantly (*p* < 0.05) within the final body weight level group (100 kg, 120 kg, and 130 kg). ^A,B^—different letters differ significantly (*p* < 0.05) between 1, 3, and 5 groups (fed with 14% CPFM during the first fattening period and with 12% CPFM during the second fattening period). ^a,b^—different letters differ significantly (*p* < 0.05) between 2, 4, and 6 groups (fed with 12% CPFM during the first fattening period and with 10% CPFM during the second fattening period).

**Table 10:** Chemical composition of the meat.

Indicators	Groups of Pigs					
	A	B	C	D	E	F
	$\bar{x}$ ± s	$\bar{x}$ ± s	$\bar{x}$ ± s	$\bar{x}$ ± s	$\bar{x}$ ± s	$\bar{x}$ ± s
Water (%)	67.78 ^A^ ± 1.50	67.51 ^a^ ± 1.54	63.05 ^B^ ± 3.78	62.80 ^b^ ± 3.45	62.31 ^B^ ± 3.09	62.04 ^b^ ± 3.74
Crude protein (%)	24.18 ^A^ ± 1.03	23.76 ^a^ ± 1.19	20.21 ^B^ ± 1.43	20.00 ^b^ ± 1.31	20.15 ^B^ ± 1.27	20.07 ^b^ ± 1.35
Crude fat (%)	6.97 ^A^ ± 1.37	7.70 ^a^ ± 1.93	15.82 ^B^ ± 5.32	16.30 ^b^ ± 4.72	16.62 ^B^ ± 4.54	16.98 ^b^ ± 4.88
Ash (%)	1.07 ^A^ ± 0.02	1.03 ^a^ ± 0.03	0.92 ^B^ ± 0.06	0.90 ^b^ ± 0.06	0.92 ^B^ ± 0.05	0.91 ^b^ ± 0.06

^A,B^—different letters differ significantly (*p* < 0.05) between 1, 3, and 5 groups (fed with 14% CPFM during the first fattening period and with 12% CPFM during the second fattening period). ^a,b^—different letters differ significantly (*p* < 0.05) between 2, 4, and 6 groups (fed with 12% CPFM during the first fattening period and with 10% CPFM during the second fattening period).

**Table 11:** Physical and chemical properties of kulens.

Indicators	Groups of Pigs					
	A	B	C	D	E	F
	$\bar{x}$ ± s	$\bar{x}$ ± s	$\bar{x}$ ± s	$\bar{x}$ ± s	$\bar{x}$ ± s	$\bar{x}$ ± s
pH	5.92 ± 0.11	5.96 ± 0.08	5.96 ± 0.16	5.92 ± 0.05	5.97 ± 0.15	5.86 ± 0.05
Water activity (a_w_)	0.87 ^A^ ± 0.01	0.84 ^a^ ± 0.03	0.81 ^B^ ± 0.03	0.79 ^b^ ± 0.02	0.79 ^B^ ± 0.03	0.83 ^ab^ ± 0.06
Color (CIE L*)	35.38 ± 1.25	35.65 ± 1.95	36.66 ± 1.67	36.18 ± 1.51	36.72 ± 1.86	36.21 ± 1.39
Color (CIE a*)	17.08 ± 0.80	17.27 ± 0.16	17.10 ± 0.99	17.57 ± 0.74	17.36 ± 1.06	17.60 ± 0.66
Color (CIE b*)	9.27 ± 1.01	9.42 ± 0.62	10.00 ± 1.23	9.80 ± 0.85	10.11 ± 1.23	9.87 ± 0.69
NaCl (%)	5.22 ± 0.12	5.18 ± 0.09	5.01 ± 0.47	5.31 ± 0.29	5.22 ± 0.43	5.42 ± 0.20
Water (%)	31.45 ± 0.56	31.01 ± 0.86	31.39 ± 3.07	32.05 ± 2.65	32.10 ± 3.20	32.61 ± 2.64
Crude protein (%)	45.94 ^A^ ± 0.19	45.74 ^a^ ± 0.71	43.89 ^B^ ± 1.63	43.39 ^b^ ± 1.15	43.59 ^B^ ± 1.73	43.00 ^b^ ± 1.09
Crude fat (%)	16.24 ^xA^ ± 0.75	17.47 ^ya^ ± 0.33	18.51 ^B^ ± 0.99	18.61 ^b^ ± 1.03	18.24 ^B^ ± 0.97	18.33 ^ab^ ± 1.04
Ash (%)	6.37 ^x^ ± 0.56	5.78 ^y^ ± 0.14	6.21 ± 0.60	5.95 ± 0.54	6.07 ± 0.62	5.97 ± 0.52

^x,y^—different letters differ significantly (*p* < 0.05) within the final body weight level group (100 kg, 120 kg, and 130 kg). ^A,B^—different letters differ significantly (*p* < 0.05) between 1, 3, and 5 groups (fed with 14% CPFM during the first fattening period and with 12% CPFM during the second fattening period). ^a,b^—different letters differ significantly (*p* < 0.05) between 2, 4, and 6 groups (fed with 12% CPFM during the first fattening period and with 10% CPFM during the second fattening period).

**Table 12:** Sensory properties of kulens.

Indicators	Groups of Pigs					
	A	B	C	D	E	F
	$\bar{x}$ ± s	$\bar{x}$ ± s	$\bar{x}$ ± s	$\bar{x}$ ± s	$\bar{x}$ ± s	$\bar{x}$ ± s
Appearance (1–5)	4.42 ± 0.36	4.55 ± 0.28	4.50 ± 0.32	4.65 ± 0.20	4.52 ± 0.38	4.40 ± 0.18
Structure (1–3)	2.67 ± 0.21	2.80 ± 0.21	2.75 ± 0.23	2.92 ± 0.24	2.76 ± 0.26	2.70 ± 0.26
Cross-section appearance (1−10)	8.30 ± 0.32	8.60 ± 0.34	8.50 ± 0.32	8.62 ± 0.25	8.42 ± 0.31	8.45 ± 0.21
Odor (1−5)	4.65 ± 0.09	4.55 ± 0.35	4.60 ± 0.30	4.65 ± 0.14	4.75 ± 0.23	4.67 ± 0.21
Taste (1−10)	8.75 ^A^ ± 0.18	8.80 ^a^ ± 0.15	9.32 ^B^ ± 0.34	9.30 ^b^ ± 0.28	9.52 ^B^ ± 0.21	9.37 ^b^ ± 0.17
General impression (1−5)	4.20 ± 0.21	4.30 ± 0.24	4.37 ± 0.31	4.45 ± 0.18	4.42 ± 1.20	4.35 ± 0.26

^A,B^—different letters differ significantly (*p* < 0.05) between 1, 3, and 5 groups (fed with 14% CPFM during the first fattening period and with 12% CPFM during the second fattening period). ^a,b^—different letters differ significantly (*p* < 0.05) between 2, 4, and 6 groups (fed with 12% CPFM during the first fattening period and with 10% CPFM during the second fattening period).

## Data Availability

The data presented in this study are available on request from the corresponding author.
